# The Crosstalk between GPR81/IGFBP6 Promotes Breast Cancer Progression by Modulating Lactate Metabolism and Oxidative Stress

**DOI:** 10.3390/antiox11020275

**Published:** 2022-01-29

**Authors:** Lucia Longhitano, Stefano Forte, Laura Orlando, Stephanie Grasso, Alessandro Barbato, Nunzio Vicario, Rosalba Parenti, Paolo Fontana, Angela M. Amorini, Giuseppe Lazzarino, Giovanni Li Volti, Michelino Di Rosa, Arcangelo Liso, Barbara Tavazzi, Giacomo Lazzarino, Daniele Tibullo

**Affiliations:** 1 Department of Biomedical and Biotechnological Sciences, University of Catania, 95123 Catania, Italy; lucialonghitano@hotmail.it (L.L.); lauraorlando2810@gmail.com (L.O.); stephanie.grasso13@gmail.com (S.G.); alessandrobarbato93@libero.it (A.B.); nunziovicario@unict.it (N.V.); parenti@unict.it (R.P.); amorini@unict.it (A.M.A.); lazzarig@unict.it (G.L.); livolti@unict.it (G.L.V.); mdirosa@unict.it (M.D.R.); d.tibullo@unict.it (D.T.); 2 IOM Ricerca Srl, 95029 Viagrande, Italy; stefano.forte@grupposamed.com (S.F.); antheafontana@libero.it (P.F.); 3 Department of Medical and Surgical Sciences, University of Foggia, 71100 Foggia, Italy; arcangelo.liso@unifg.it; 4 UniCamillus—Saint Camillus International University of Health Sciences, Via di Sant’Alessandro 8, 00131 Rome, Italy; giacomo.lazzarino@unicamillus.org

**Keywords:** lactate, IGFBP6, GPR81, oxidative stress, breast cancer

## Abstract

Breast cancer is the most frequent tumor and the leading cause of cancer deaths in women. In recent years, lactate metabolism and, in particular, its receptor GPR81 have been shown to play a vital role in cancer biology. GPR81 is upregulated in breast cancer and promotes tumor growth by tumor cell-derived lactate. Therefore, the search for possible crosstalk and the involvement of new molecules capable of generating this pathology is always in continuous development. In this study, the relationship between GPR81 and IGFBP6 protein in tumor growth and oxidative stress in the human breast cancer cell line MDA-MB-231 was studied. Cells were treated with lactate or the GPR81 receptor agonist and antagonist 3,5-DHBA and 3-OBA, respectively. In addition, oxidative stress and proliferation were also evaluated in cells challenged with the recombinant IGFBP6 protein. Our data showed that lactate induced cell proliferation and wound healing of the MDA-231 breast cancer cell through the overexpression of both the lactate receptor GPR81 and IGFBP6. The increase in IGFBP6 was able, in turn, to improve the mitochondrial fitness and redox state, as suggested by the reduced levels of mitochondrial ROS production after IGFBP6 treatment, presumably mediated by the increase in the ROS detoxifying genes HMOX1, GSTK1 and NQO1. In conclusion, our data highlight a novel axis between GPR81 and IGFBP6 in MDA-231 cells able to modulate lactate metabolism and oxidative stress. This complex signaling may represent a new therapeutic target for breast cancer.

## 1. Introduction

Breast cancer is the most common cancer among women, and is the second most frequently occurring cancer worldwide among newly diagnosed cancers [1]. It is a heterogeneous disease defined by several activating mutations, epigenetic modifications and aberrant signaling pathways. Compared to other types of cancers, such as EGFR-mutated lung cancer and BRAF-mutated melanoma, precision medicine in breast cancer is still in its infancy, despite the earlier identification of targets such as ER and HER2 [2]. Therefore, although the use of adjuvant chemotherapeutic agents and hormonal agents has improved breast cancer mortality, the long-term survival of breast cancer patients is rather low and still currently represents a major clinical challenge [3].

Tumors have a great need for bioenergy to maintain cell growth and proliferation. The hypoxic, glucose-poor tumor microenvironment requires cancer cells to alter their metabolic patterns to adapt to these conditions [4]. A high rate of glycolysis, with decreased metabolism of mitochondrial oxidative phosphorylation (OXPHOS), is a universal phenomenon in cancer cells [5,6], causing lactate accumulation within the cells. However, glycolysis and OXPHOS coexist in tumor cells and facilitate tumorigenesis [7]. Moreover, high lactate production from cancer cell metabolism facilitates tumor progression and metastasis [8,9,10], and suppresses both innate and adaptive immune cells in the tumor microenvironment (TME) [11,12,13,14].

The recent discovery of a cell surface receptor for lactate in mammalian cells represents a new era in terms of understanding the exact connection of the Warburg effect to cancer; in fact, lactate transmits pro-tumor signals through its G-protein-coupled receptor 81 (GPR81), highly expressed in breast, pancreatic and cervical carcinomas, whilst negligibly expressed in the benign epithelium of these tissues [8].

Cell lactate uptake primarily occurs via monocarboxylate transporter 1 (MCT1), which has a high binding capacity for lactate [15]. The intracellular lactate increase activates GPR81, which inhibits the conversion of ATP to cAMP [16]. Roland et al. [8] demonstrated that GPR81, by altering the expression of MCT1 and MCT4 in the presence of lactate and glucose, is involved in the overall regulation of lactate transport mechanisms of tumor cells. A further confirmation has been shown by the decreased sensitivity of tumor cells to lactate when silencing GPR81 [17], suppressing tumor proliferation and metastasis [8,16]. Although obtained in a model of brain ischemia, it has been found that 3-hydroxy-butyrate (3-OBA) acts as a putative GPR81 antagonist and can effectively be used to block the lactate/GPR81 pathway, with the protection of cells from glucose deprivation [18].

The role of some genes in tumor growth is now the focus of research. The insulin-like growth factor binding protein 6 (IGFBP6) gene plays an important role in the pathogenesis of many malignancies, and is actively involved in proliferation, wound healing and the survival of tumor cells [19]. The protein product of IGFBP6 is a secreted *O*-linked glycoprotein that binds to insulin-like growth factors (IGFs), preventing their action on cells [19,20]. The main mechanism of IGFBP6 occurs through IGF-2 binding and the consequent inhibition of IGF-2 activity [21]. However, IGFBP6 is capable of interacting with proteins other than IGF-2, thus affecting cell functions through IGF-2-independent mechanisms [21]. The role of the IGFBP6 gene in cancer development and progression has not yet been fully elucidated. According to some data, expression of the IGFBP6 gene is a reliable predictor for the recurrence of breast, stomach and nasopharyngeal cancers. Conversely, other studies have shown that the level of IGFBP6 is lower in tumor tissues than in normal cells, implying an antitumor effect of IGFBP6 [22].

In order to better clarify their roles in tumor growth, the aim of the present study was to evaluate the lactate-mediated crosstalk of GPR81 and IGFBP6 through the determination of cell proliferation, wound healing, mitochondrial oxidative metabolism and oxidative stress levels in human breast cancer cells (MDA-MB-231) in vitro.

## 2. Materials and Methods

### 2.1. Cell Culture and Pharmacological Treatment

Human breast cancer cell line (MDA-MB-231) was purchased from ATCC Company (Milan, Italy). Cells were suspended in DMEM-F12 (Gibco, cat. no. 11965092) culture medium containing 10% fetal bovine serum (FBS, Gibco, cat. no. 10082147), 100 U/mL penicillin and 100 U/mL streptomycin (Gibco, cat. no. 15070063). At 80% confluency, cells were passaged using trypsin-EDTA solution (0.05% trypsin and 0.02% EDTA, Gibco, cat. no. 25300054). Lactate, 3,5-dihydroxybenzoic acid (3-5-DHBA), 3-hydroxybutyric acid (3-OBA) and IGFBP6 (Sigma–Aldrich, Milan, Italy) were added to cell culture at final concentrations of 20 mM, 150 μM, 3 mM and 400–800 ng/mL, respectively, dissolved in cell culture media for different times (4 to 48 h). Same amount of cell culture media was used for control cultures.

### 2.2. Real-Time Monitoring of Cell Proliferation

xCELLigence experiments were performed using the RTCA (Real-Time Cell Analyzer) DP (Dual Plate) instrument according to manufacturers’ instructions (Roche Applied Science, Mannheim, Germany; ACEA Biosciences, San Diego, CA, USA). The RTCA DP instrument includes the following three main components: (i) RTCA DP Analyzer, which is placed inside a humidified incubator maintained at 37 °C and 5% CO_2_, (ii) RTCA Control Unit with RTCA Software preinstalled and (iii) E-Plate 16 for proliferation assay. First, the optimal seeding number was determined by cell titration and growth experiments. After seeding the optimal cell number (3000 cells/well), cells were treated and automatically monitored every 15 min for 48 h. Optimal cell number was determined in a preliminary set of experiments (data not shown) to obtain a significant cell index value and constant cell growth during the entire duration of the experiment.

### 2.3. Real-Time PCR for Gene Expression Analysis

RNA was extracted by Trizol^®^ reagent (category no. 15596026, Invitrogen, Carlsbad, CA, USA). The first-strand cDNA was then synthesized with High-Capacity cDNA Reverse Transcription kit (category no. 4368814, Applied Biosystems, Foster City, CA, USA). High cDNA quality was checked, taking into consideration the housekeeping gene Ct values. Quantitative real-time PCR was performed in Step-One Fast Real-Time PCR system, Applied Biosystems, using SYBR Green PCR MasterMix (category no. 4309155, Life Technologies, Monza, Italy). The specific PCR products were detected by the fluorescence of SYBR Green, the double-stranded DNA binding dye. Primers were designed using BLAST^®^ (Basic Local Alignment Search Tool, NBCI, NIH), considering the shortest amplicon proposed (primers’ sequences are shown in Table 1), and β-actin was used as the housekeeping gene. Primers were purchased from Metabion International AG (Planneg, Germany). The relative mRNA expression level was calculated by the threshold cycle (Ct) value of each PCR product and normalized with β-actin by using a comparative 2^−ΔΔCt^ method.

### 2.4. Western Blot Analysis

Briefly, for Western blot analysis, 30 μg of protein was loaded onto a 12% polyacrylamide gel MiniPROTEAN^®^ TGXTM (BIO-RAD, Milan, Italy) followed by electrotransfer to nitrocellulose membrane TransBlot^®^ TurboTM (BIO-RAD, Milan, Italy) using TransBlot^®^ SE Semi-Dry Transfer Cell (BIO- RAD, Milan, Italy). Subsequently, membrane was blocked in Odyssey Blocking Buffer (Licor, Milan, Italy) for 1 h at room temperature. After blocking, membrane was washed in phosphate-buffered saline (PBS) three times for 5 min and incubated with primary antibodies against GPR81 (1:1000), IGFBP6 (1:500) and β-actin (1:1000) (anti-mouse, Cat. No. 4967S, Cell Signalling Technology, Milan, Italy), overnight at 4 °C. The next day, membranes were washed in PBS three times for 5 min and incubated with infrared anti-mouse IRDye800CW (1:5000) and anti-rabbit IRDye700CW secondary antibodies (1:5000) in PBS/0.5% Tween-20 for 1 h at room temperature. All antibodies were diluted in Odyssey Blocking Buffer. The blots were visualized using Odyssey Infrared Imaging Scanner (Licor, Milan, Italy), and protein levels were quantified by densitometric analysis. Data were normalized to β-actin expression.

### 2.5. Wound Healing Assay

Cell proliferation was assessed by a “wound healing” assay. Cells were seeded separately in 6-well dishes and cultured until confluence. Cells were scraped with a 200 μL micropipette tip and monitored at 0 h and 24 h. The uncovered wound area was measured and quantified at different intervals with ImageJ 1.37v (NIH). The experiments were performed in quadruplicates.

### 2.6. Clonogenic Assay

Colony assays were performed by seeding cells in 6-well plates at low density (5000 cells/well) and allowing growth for 10 days. Colonies were fixed and stained with crystal violet. The experiments were performed in quadruplicates.

### 2.7. Lactate Concentration Assay

The spectrophotometric determination of lactate was carried out using an Agilent 89090A spectrophotometer (Agilent Technologies, Santa Clara, CA, USA). Briefly, the reaction mixture contained 100 mM Tris-HCl, 1.5 mM N-ethyl-N-2-hydroxy-3-sulfopropyl-3-methylalanine, 1.7 mM 4-aminoantipyrine, and 5 IU horseradish peroxidase. Fifty microliters of serum was added to the mixture, left to stand for 5 min and read at 545 nm wavelength. The reaction was started with the addition of 5 IU of lactate oxidase to the cuvette (finale volume = 1 mL) and it was considered finished when no change in absorbance was recorded for at least 3 min. To calculate lactate in samples, the difference in absorbance at 545 nm wavelength (Δabs) of each sample was interpolated with a calibration curve obtained by plotting Δabs measured in standard solutions of lactate with increasing known concentrations.

### 2.8. Flow Cytometry

To determine the intracellular ROS levels, cells were stained with 2′,7′-dichlorodihydrofluorescein acetate (H2-DCF; Sigma-Aldrich), and fluorescence intensity was measured according to the fluorescence detection conditions of FITC using the MACSQuant Analyzer (Miltenyi Biotech). A membrane potential probe, the 3,3′-diethyloxacarbocyanine iodide (DiOC2(3)), was used to evaluate the mitochondrial membrane potential. Cells were incubated with 10 μM DiOC2(3) (Thermo Fisher Scientific, Milan, Italy) for 30 min at 37 °C, washed twice, resuspended in PBS and analyzed by flow cytometry through the detection of the green fluorescence intensity of DiOC2(3).

### 2.9. Statistical Analysis

For statistical analyses, a two-tailed unpaired Student’s t-test was used for comparison of *n* = 2 groups. For comparison of *n* ≥ 3 groups, one-way analysis of variance (ANOVA), followed by Bonferroni post hoc test for multiple comparisons was used. Data are presented as the mean ± SD of biological replicates and are shown as scattered dot plots or box and whisker plots. Data analysis was performed using GraphPad Prism software version 5.00. A value of *p* < 0.05 was considered statistically significant and symbols used to indicate statistical differences are described in figure legends.

## 3. Results

### 3.1. GPR81 Stimulation Promotes Breast Cancer Cell Growth and Modulates Mitochondrial Metabolism Gene Expression

**Figure 1:**
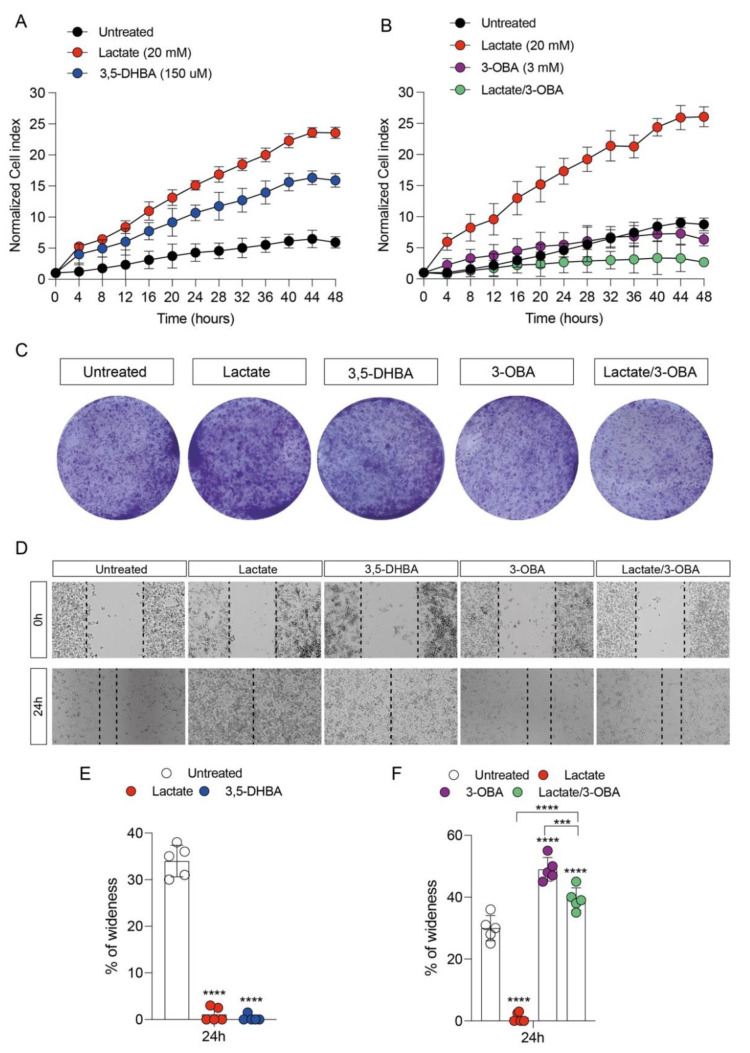
GPR81 stimulation promotes breast cancer cell growth. Effect of lactate (20 mM), 3,5-DHBA (150 μM, GPR81 agonist) and 3-OBA (3 mM, GPR81 antagonist) on cell proliferation (**A**,**B**), colony formation capacity (**C**) and wound healing (**D**–**F**). Data are expressed as mean ± SD of at least four independent experiments. (*** *p* < 0.001; **** *p* < 0.0001).

**Figure 2:**
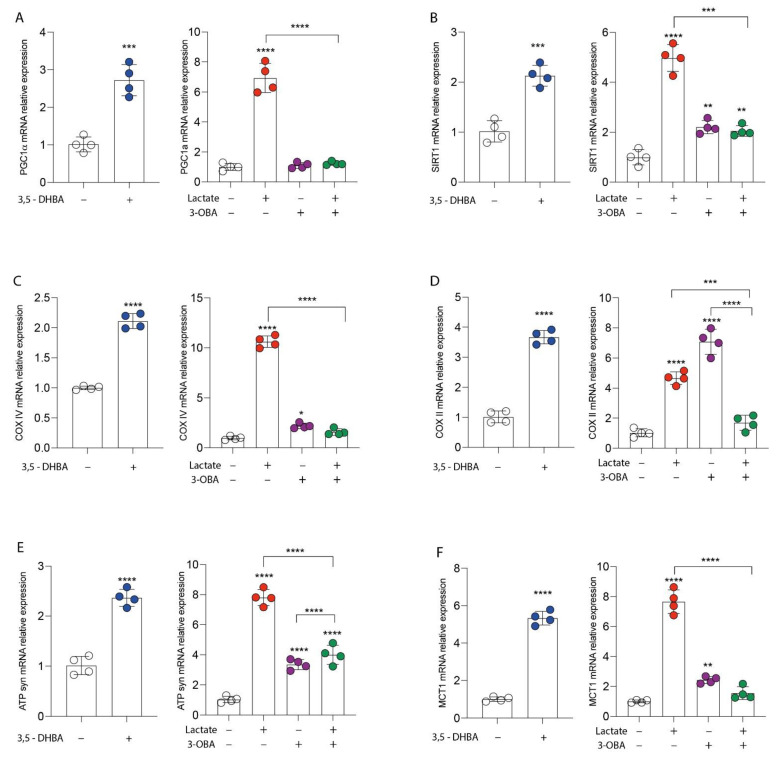
GPR81 modulates mitochondrial metabolism gene expression. Evaluation of relative mRNA expression levels of (**A**) PGC1alpha, (**B**) SIRT1, (**C**) COX IV, (**D**) COX II, (**E**) ATPsyn, (**F**) MCT1, following 24 h of lactate (20 mM), 3,5-DHBA (150 μM) and 3-OBA (3 mM) exposition, analyzed by real-time PCR. The calculated value of 2^−ΔΔCt^ in untreated controls is 1. Data are expressed as mean ± SD of at least four independent experiments. * *p* < 0.05; ** *p* < 0.005; *** *p* < 0.001; **** *p* < 0.0001.

We first studied the effect of lactate signaling, through its GPR81 receptor activation, in breast cancer cell proliferation. We exposed MDA-MB-231 cells to 20 mM of lactate and to a selective agonist of the GPR81 receptor (3,5-DHBA 150 μM), and we analyzed real-time cell proliferation, colony formation capacity and wound healing. Our results showed that both lactate and 3,5-DHBA were able to induce a significant increase in cell proliferation (Figure 1A) and colony formation (Figure 1C) compared to untreated cells. To further confirm the effect of lactate on cell proliferation via activation of the GPR81 receptor, we also exposed MDA-MB-231 cells to the selective receptor antagonist (3-OBA, 3 mM) and to the lactate/3-OBA combination. Interestingly, treatment with 3-OBA alone did not result in significant effects compared to control cells, while lactate/3-OBA co-treatment significantly reduced cell proliferation, as indicated by the normalized cell index (Figure 1B). As shown in Figure 1C, this effect was further confirmed, as the lactate/3-OBA co-treatment also significantly reduced the number of colonies compared to their controls. We then tested whether the activation of lactate and its receptor affects the wound healing of breast cancer cells. Interestingly, we observed a reduced percentage of wideness of the scratch assay test at 24 h in lactate and 3,5-DHBA-exposed cells (Figure 1D,E). We also confirmed the effects of GPR81 inhibition through 3-OBA on cell proliferation, finding a significantly increased percentage of wideness of the scratch assay test at 24 h in 3-OBA and lactate/3-OBA-treated cells (Figure 1D–F). In an attempt to show that GPR81 activation is a positive modulator of cell proliferation and wound healing, we investigated whether receptor stimulation and/or inhibition was involved in the modulation of genes involved in the mitochondrial metabolism pathway. Our data showed that both lactate and 3,5-DHBA exposure for 24 h result in a significant increase in PPARG coactivator 1 alpha (*PGC1α*) (Figure 2A) and sirtuin *1* (*SIRT1*) (Figure 2B) mRNA expression levels, involved in mitochondrial biogenesis, and in cytochrome c oxidase subunit 4 (*COX IV*) (Figure 2C), *COX II* (Figure 2D) and ATP synthase (*ATPsyn*) (Figure 2E) mRNA expression levels, involved in OXPHOS. Interestingly, up-regulation of the aforementioned genes was blocked by the lactate/3-OBA co-treatment, suggesting that the activation of the GPR81 receptor modulates the mitochondrial metabolism of the tested cells (Figure 2A–E). Since MCT1 and GPR81 are used, respectively, by oxidative cells for the uptake of extracellular lactate, and by tumor cells to modulate lactate-induced changes in cell functions, we analyzed the expression of MCT1 mRNA levels after exposure to both lactate and its receptor agonist. Up-regulation of MCT1 was found when these compounds were added separately, whilst co-treatment produced evident downregulation (Figure 2F).

### 3.2. GPR81/IGFBP6 Axis Activation in Breast Cancer Cells

**Figure 3:**
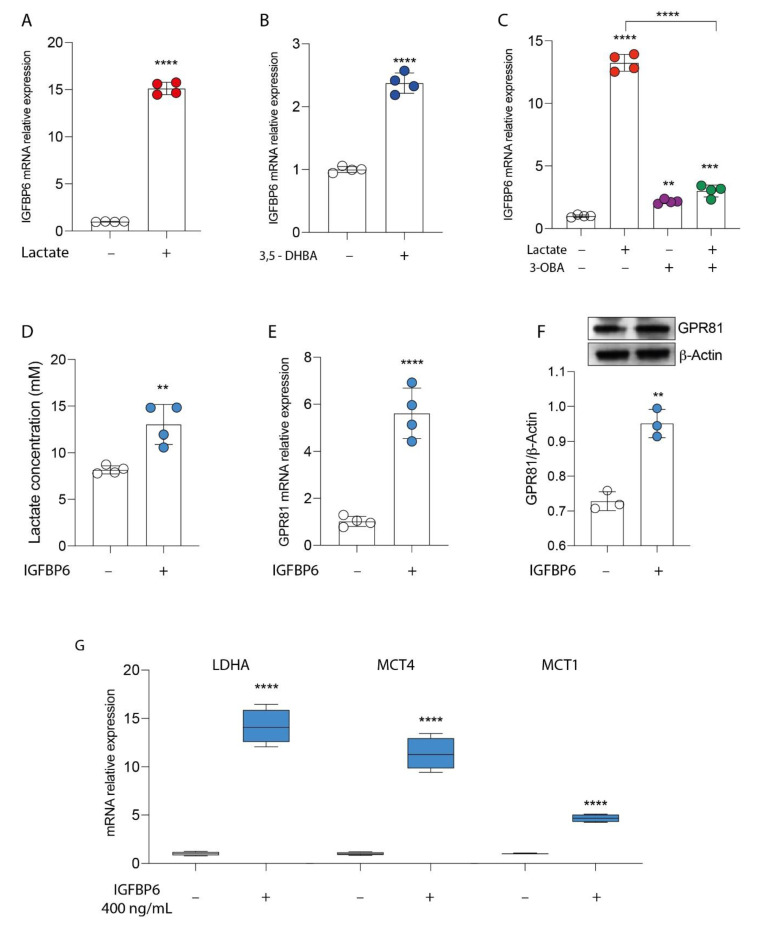
GPR81/IGFBP6 axis in breast cancer cells. Effect of lactate (20 mM) (**A**), 3,5-DHBA (150 μM) (**B**) and 3-OBA (3 mM) (**C**) on IGFBP6 mRNA expression levels, following 24 h of treatment. Effect of IGFBP6 treatment (800 ng/mL) on lactate production (**D**), GPR81 mRNA expression levels (**E**) and GPR81 protein expression (**F**), following 24 h of treatment. Evaluation of relative mRNA expression levels of LDHA, MCT4 and MCT1 (**G**) in IGFBP6-exposed (400 ng/mL) cells. The calculated value of 2^-ΔΔCt^ in untreated controls is 1. Data are expressed as mean ± SD of at least four independent experiments. ** *p* < 0.005; *** *p* < 0.001; **** *p* < 0.0001.

Given the evidence of cell modulation exerted by lactate receptor activation, we tried to link the molecular mechanisms underlying these phenomena with the expression of IGFBP6. To this end, we first analyzed whether lactate increased the expression of IGFBP6. As shown in Figure 3A,B, treatment with lactate in MDA-MB-231 cells resulted in a significant increase in the mRNA relative expression of IGFBP6 (Figure 3A) compared to the untreated control cells. The same effect was found after receptor stimulation (Figure 3B), and, again, the lactate effect was significantly reversed by adding the receptor inhibitor 3-OBA (Figure 3C). Then, to investigate the existence of an axis between lactate, MCT1-4 receptors and IGFBP6, the breast cancer cells were exposed to the recombinant protein IGFBP6 for 24 h. Surprisingly, IGFBP6 treatment induced a significant increase in lactate production in the supernatant of MDA-MB-231 cells (Figure 3D), as well as a significant increase in both mRNA (Figure 3E) and GPR81 protein expression levels (Figure 3F), compared to the corresponding values found in the control cells. To further confirm these data, we performed real-time PCR analysis to evaluate the expression of the LDHA enzyme and lactate transporters MCT1 and MCT4.

Consistently, our data showed that the treatment of breast cancer cells with IGFBP6 significantly induced an increase in the mRNA expression of the LDHA, MCT1 and MCT4 genes (Figure 3G).

### 3.3. IGFBP6 Recapitulates Lactate/GPR81 Agonist Effects on MDA-MB-231 Cells

**Figure 4:**
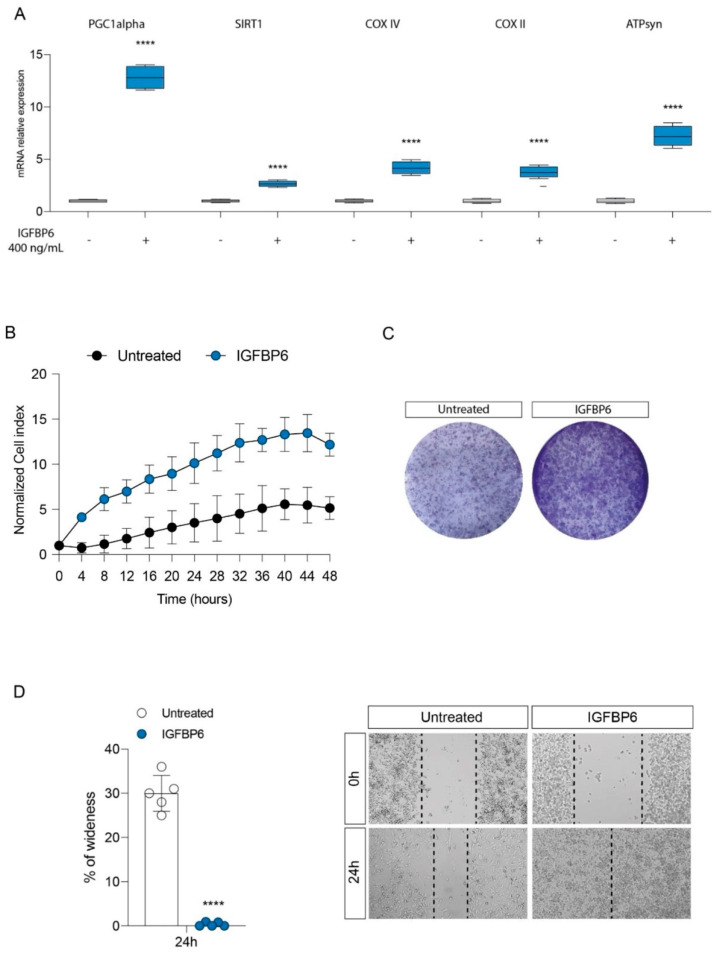
IGFBP6 modulates mitochondrial metabolism and promotes breast cancer cell proliferation. Evaluation of relative mRNA expression levels of PGC1 alpha, SIRT1, COX IV, COX II and ATPsyn (**A**), following 24 h of IGFBP6 (400 ng/mL) treatment. The calculated value of 2^−ΔΔCt^ in untreated controls is 1. Effect of IGFBP6 exposure (800 ng/mL) on cell proliferation (**B**), colony formation capacity (**C**) and wound healing (**D**). Data are expressed as mean ± SD of at least four independent experiments. **** *p* < 0.0001.

Since lactate, through the activation of the GPR81 receptor, promotes tumor proliferation and wound healing, and the switch towards the oxidative phenotype in MDA-MB-231 cells, and given the stimulation of IGFBP6 production after exposure to lactate or an antagonist, and vice versa, we evaluated the effect of direct exposure to IGFBP6 on the expression of the genes involved in the pathway of mitochondrial metabolism, and on the proliferation of the cells under examination. Consistently with the data reported above, we have shown that even direct exposure to IGFBP6 resulted in significant up-regulation of the genes involved in mitochondrial biogenesis, *PGC1α* and *SIRT1*, and significant up-regulation of the OXPHOS genes, *COX IV*, *COX II* and *ATP syn* (Figure 4A). Furthermore, an increase in cell proliferation, as shown by the cell index value, was shown in IGFBP6-treated cells compared to untreated cells (Figure 4B), as well as an increase in colony-forming capacity (Figure 4C). Finally, scratch analysis revealed that IGFBP6 in MDA-MB-231 cells resulted in an increase in percentage wound closure at 24 h post treatment compared to untreated cells (Figure 4D).

### 3.4. IGFBP6 Induces an Antioxidant Response in MDA-MB-231 Cells

**Figure 5:**
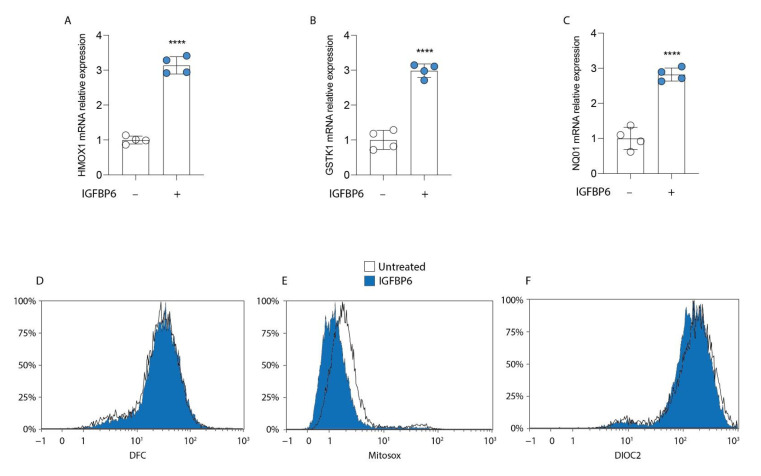
IGFBP6 induces an antioxidant response in MDA-MB-231 cells. Evaluation of relative mRNA expression levels of HMOX1 (**A**), GSTK1 (**B**) and NQ01 (**C**), following 24 h of IGFBP6 (800 ng/mL) treatment. The calculated value of 2^−ΔΔCt^ in untreated controls is 1. Data are expressed as mean ± SD of at least four independent experiments. **** *p* < 0.0001. Representative plots of ROS (**D**) and mitochondrial ROS (**E**) production using DCF and MitoSox probe, following 24 h of IGFBP6 exposure. Representative plot of mitochondrial membrane potential (**F**) evaluated with DiOC2 staining, following 24 h of IGFBP6 treatment. Plots are representative of at least four independent experiments.

Since IGFBP6 is involved in MDA-MB-231 cell proliferation and the increase in mitochondrial metabolism, we analyzed the cellular redox status of these cells during IGFBP6 exposure. Interestingly, compared to the control untreated cells, the breast cancer cells reacted to 24 h of IGFBP6 exposure by inducing significant up-regulation of antioxidant-related genes, such as heme oxygenase 1 (*HMOX1*; Figure 5A), glutathione S-transferase kappa 1 (*GSTK1*; Figure 5B), and NAD(P)H dehydrogenase (quinone) 1 (*NQ01*; Figure 5C). These data were confirmed by cytofluorimetric analysis, which showed a decrease in MitoSox staining in IGFBP6-treated cells (Figure 5E), a decrease in DiOC2 staining intensity in IGFBP6-treated cells (Figure 5F), and no difference in ROS production (Figure 5D).

## 4. Discussion

The results obtained in the present study allowed us to better depict the role of lactate in breast cancer cells and the mechanisms underpinning the lactate-mediated changes in their metabolism and functions. Our data confirmed the capacity of lactate to induce cell proliferation and wound healing in breast cancer cells, and, for first time to the best of our knowledge, they showed that these effects occurred through a complex mechanism involving the following: (i) stimulation of the lactate transporter MCT1-4; (ii) transduction of the signal through the increase in GPR81 expression; (iii) change in cell metabolism and ROS detoxifying defenses through the increase in IGFBP6 expression.

In the last few years, the role of GPR81 has increasingly been studied in all those pathophysiological conditions activated by lactate, particularly in the neurological and oncological fields [16,23,24]. In several cancer cell types, including those of colon, breast, lung, cervical, and pancreatic cancer, GPR81 is highly expressed, suggesting a potential broad role of GPR81 in tumorigenesis. Moreover, GPR81 levels also correlated with the rates of cancer cell proliferation and metastasis in vivo, and were also elevated in xenografted cells. These data suggest that the expression of GPR81 is fundamental for cancer cells within the tumor microenvironment. Altering the cellular metabolism of the tumor microenvironment is a hallmark of cancer, and lactate metabolism has increasingly been recognized to have a critical role in tumor cell survival. In the tumor microenvironment, increased lactate levels significantly activate GPR81, mediating the activation of cell survival signaling [25]. Among the most interesting biological processes reshaping cell metabolism, those that recap developmental processes through IGFBP6 involvement hold great potential to understand tumor-related biological processes, such as cell proliferation, wound healing, senescence, and changes in metabolism [26].

The present data highlighted the relevance of the lactate-induced GPR81/IGFBP6 crosstalk in tumor progression in MDA-MB-231 breast cancer cells. Firstly, we observed that the treatment of cancer cells with lactate resulted in a significant increase in cell proliferation by activating the GPR81 receptor. This strongly corroborates the notion of GPR81 involvement in the main biological processes guiding and supporting carcinogenesis. To confirm the involvement of the lactate receptor, we used the selective agonist of the GPR81 receptor 3,5-dihydroxybenzoic acid (3,5-DHBA). It has been observed that 3,5-DHBA, at the cellular level, mimics the effects induced by lactate (activation of the GPR81 receptor and increase in the expression of the lactate transporter MCT1-4). Consistent with our data, Lee et al. showed that GPR81 is a putative tumor-promoting gene that promotes angiogenesis and the survival of breast cancer cells in the tumor microenvironment [25].

In addition, we found a greater expression of genes involved in mitochondrial biogenesis, suggesting an increase in the mitochondrial fitness of the cell line under examination. To confirm these results, we analyzed the effects of concomitant treatment with lactate and a selective antagonist of the GPR81 receptor (3-OBA). Interestingly, our data showed that the lactate-induced effects were reversed by 3-OBA treatment. Additionally, modulation of the gene expression of IGFBP6 (increased following treatment with 3,5-DHBA and reduced by 3-OBA treatment) was also observed. The increase in IGFBP6 expression produced a significant improvement in the mitochondrial fitness and redox state. The reduced levels of mitochondrial ROS after IGFPB-6 treatment were presumably due to the increase in the ROS detoxifying genes HMOX1, GSTK1 and NQO1. This latter phenomenon may allow cancer cells to have higher resistance towards agents that induce mitochondrial stress, including the drugs and therapies most used to treat cancer in the clinical setting. Mitochondria produce large quantities of ROS, which are functional for signaling tumor proliferation, survival and metastatic processes. To compensate for the high rate of mitochondrial ROS production, cancer cells have evolved adaptive mechanisms to augment their antioxidant systems and to target ROS activation pathways that are useful for tumor cell adaptation to environmental changes and drug resistance [27,28,29].

Based on these observations, we hypothesized a correlation between the IGFBP6 protein and the lactate signaling pathway. Our results confirmed that treatment with IGFBP6 resulted in an increase in cell proliferation, wound healing, and gene expression of lactate transporters (MCT1 and 4) and the GPR81 receptor.

**Figure 6:**
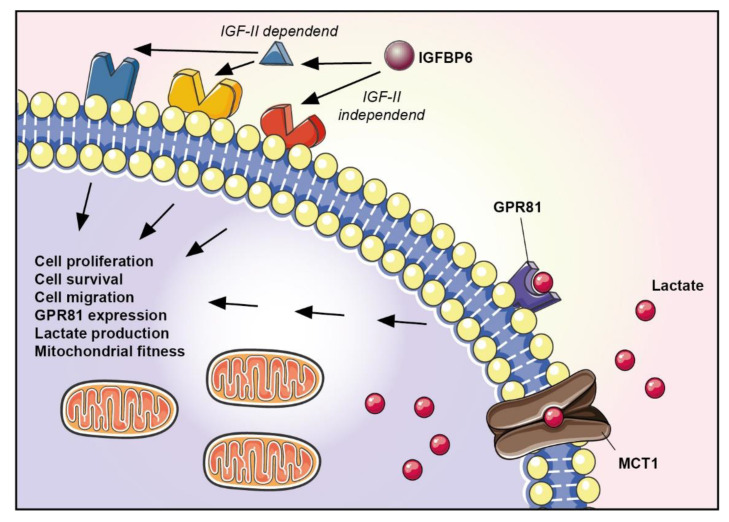
Graphical representation of GPR81 and IGFBP6 axis.

In conclusion, our data support the role of lactate metabolism in breast cancer, highlighting a new pathway activated by glucose metabolism in the tumor microenvironment, i.e., the activation of GPR81/IGFBP6 crosstalk (Figure 6), which may represent a new therapeutic target in breast cancer. Future studies focused on in vitro and in vivo models of breast cancers are needed to highlight the role of the GPR81/IGFBP6 axis in additional cell lines, homocellular the heterocellular communication, and metabolic signaling of the tumor microenvironment.

## Figures and Tables

**Table 1:** Gene of interest.

Gene of Interest	Forward Primer (5′ → 3′)	Reverse Primer (5′ → 3′)
PGC1α	ATGAAGGGTACTTTTCTGCCCC	GGTCTTCACCAACCAGAGCA
SIRT1	AGGCCACGGATAGGTCCATA	GTGGAGGTATTGTTTCCGGC
COX IV	GCGGCAGAATGTTGGCTAC	AGACAGGTGCTTGACATGGG
COX II	ACGACCTCGATGTTGGATCA	ATCATTTACGGGGGAAGGCG
ATPsyn	CCGCCTTCCGCGGTATAATC	ATGTACGCGGGCAATACCAT
MCT1	TGTTGTTGCAAATGGAGTGT	AAGTCGATAATTGATGCCCATGCCAA
MCT4	TATCCAGATCTACCTCACCAC	GGCCTGGCAAAGATGTCGATGA
IGFBP6	GACCAGGAAAGAATGTGAAAGGA	GCTCTGCCAATTGACTTTCCTTAG
HCAR1	TTCGTATTTGGTGGCAGGCA	TTTCGAGGGGTCCAGGTACA
LDHA		
HMOX1	AAGACTGCGTTCCTGCTCAAC	AAAGCCCTACAGCAACTGTCG
GSTK1	CTGGGCTTCGAGATCCTGTG	GGCAGACAAACTTCCACTGTC
NQ01		
β-Actin	CCTTTGCCGATCCGCCG	AACATGATCTGGGTCATCTTCTCGC

## Data Availability

All of the data is contained within the article.
