# Correction: Longhitano et al. The Crosstalk between GPR81/IGFBP6 Promotes Breast Cancer Progression by Modulating Lactate Metabolism and Oxidative Stress. *Antioxidants* 2022, *11*, 275

**DOI:** 10.3390/antiox14060687

**Published:** 2025-06-05

**Authors:** Lucia Longhitano, Stefano Forte, Laura Orlando, Stephanie Grasso, Alessandro Barbato, Nunzio Vicario, Rosalba Parenti, Paolo Fontana, Angela M. Amorini, Giuseppe Lazzarino, Giovanni Li Volti, Michelino Di Rosa, Arcangelo Liso, Barbara Tavazzi, Giacomo Lazzarino, Daniele Tibullo

**Affiliations:** 1 Department of Biomedical and Biotechnological Sciences, University of Catania, 95123 Catania, Italy; lucialonghitano@hotmail.it (L.L.); lauraorlando2810@gmail.com (L.O.); stephanie.grasso13@gmail.com (S.G.); alessandrobarbato93@libero.it (A.B.); nunziovicario@unict.it (N.V.); parenti@unict.it (R.P.); amorini@unict.it (A.M.A.); lazzarig@unict.it (G.L.); mdirosa@unict.it (M.D.R.); d.tibullo@unict.it (D.T.); 2 IOM Ricerca Srl, 95029 Viagrande, Italy; stefano.forte@grupposamed.com (S.F.); antheafontana@libero.it (P.F.); 3 Department of Medical and Surgical Sciences, University of Foggia, 71100 Foggia, Italy; arcangelo.liso@unifg.it; 4 UniCamillus—Saint Camillus International University of Health Sciences, Via di Sant’Alessandro 8, 00131 Rome, Italy; giacomo.lazzarino@unicamillus.org

In the original publication, there was a mistake in Figure 4C as published [1]. The corrected Figure 4 appears below. The authors apologize for any inconvenience caused and state that the scientific conclusions are unaffected. This correction was approved by the Academic Editor. The original publication has also been updated.

**Figure 4:**
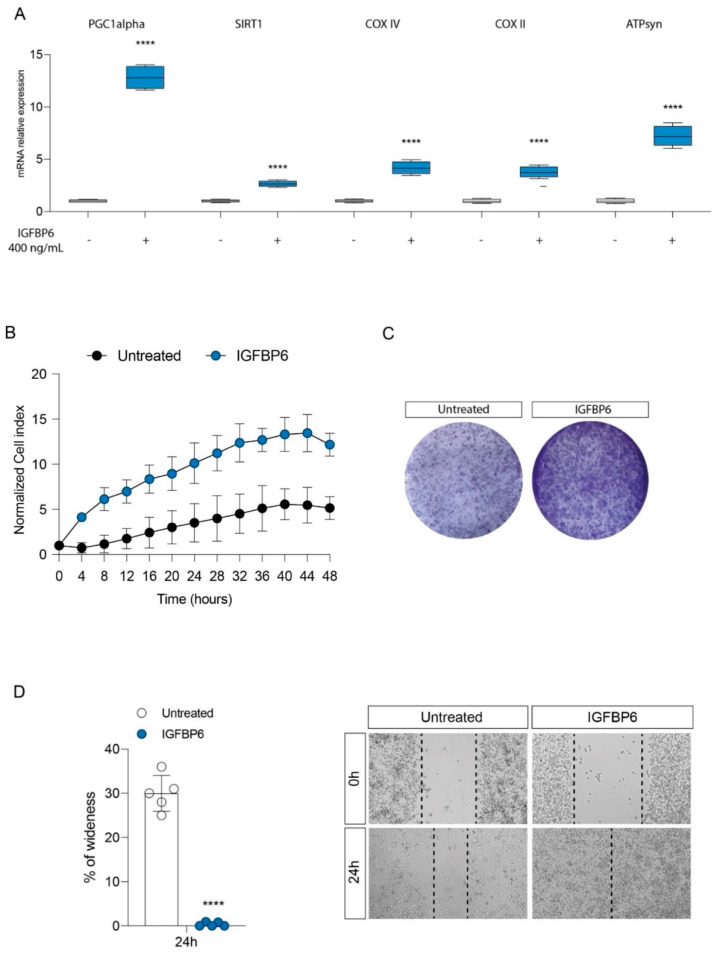
IGFBP6 modulates mitochondrial metabolism and promotes breast cancer cell proliferation. Evaluation of relative mRNA expression levels of PGC1 alpha, SIRT1, COX IV, COX II and ATPsyn (**A**), following 24 h of IGFBP6 (400 ng/mL) treatment. The calculated value of 2^−ΔΔCt^ in untreated controls is 1. Effect of IGFBP6 exposure (800 ng/mL) on cell proliferation (**B**), colony formation capacity (**C**) and wound healing (**D**). Data are expressed as mean ± SD of at least four independent experiments. **** *p* < 0.0001.
